# Impact of a refined nursing model combined with early exercise rehabilitation on patients with chronic heart failure under a risk assessment strategy

**DOI:** 10.3389/fcvm.2025.1638025

**Published:** 2025-10-08

**Authors:** Yan Sun, Haiqin Jin, Lingsha Wu

**Affiliations:** Department of Cardiology, The Second Hospital of Jiaxing, Jiaxing, Zhejiang, China

**Keywords:** risk assessment strategy, refined care, early exercise rehabilitation, chronic heart failure, cardiac function, quality of life

## Abstract

**Background:**

The treatment focus for patients with chronic heart failure (CHF) remains on acute resuscitation and maintenance during the stabilization phase. Despite significant advances in CHF management, treatment outcomes and disease control remain suboptimal. This study investigates the impact of a refined nursing model incorporating risk assessment strategies, combined with early exercise rehabilitation, on patients with CHF.

**Method:**

Ninety cases of patients with CHF admitted to our hospital between February 2024 and November 2024 were selected and divided into the control group and the study group according to the randomized numerical table method, with 45 cases in each group. The control group carried out routine nursing care under a traditional model, and the study group applied a refined nursing model under a risk assessment strategy, combined with early exercise rehabilitation for nursing care, on the basis of the traditional model.

**Result:**

The left ventricular ejection fractions (LVEFs) of CHF patients in the study group were significantly higher after cardiac ultrasound care compared with the control group, B-type natriuretic peptide was significantly lower, and 6-minute walk distance (6-MWD) was significantly longer. Walk distance (6-MWD) was significantly prolonged. The difference between the two groups was statistically significant (*t* = 2.172, *P* = 0.033; *t* = 2.097, *P* = 0.039; *t* = −4.594, *P* < 0.001). After care, patients in the study group had significantly higher scores on the activity of daily living scale and significantly lower scores on the Minnesota living with heart failure questionnaire than those in the control group. The difference between the two groups was statistically significant (*t* = −4.027, *P* < 0.001; *t* = 2.198, *P* = 0.031).

**Conclusion:**

A refined nursing model under a risk assessment strategy, combined with early exercise rehabilitation nursing, can significantly improve the cardiac function of patients with chronic heart failure and also the quality of life of such patients.

## 1 Introduction

Chronic heart failure (CHF) is a complex group of clinical syndromes characterized by impaired ventricular filling or ejection capacity due to any structural or functional cardiac abnormality, mainly manifested by dyspnea, weakness (limited activity tolerance), and fluid retention (pulmonary stasis and peripheral edema), and it is an end-stage manifestation of cardiovascular disease and the most prominent cause of death (1). Patients with CHF are becoming increasingly younger, and their susceptibility to recurrent episodes, high morbidity, mortality, and rehospitalization, coupled with symptoms such as dyspnea, and the economic pressure of high medical bills, lead to a decline in their quality of life (2–6). Despite significant advances in the treatment of CHF, outcomes and disease control are still not optimal because the focus of treatment for patients with CHF remains on resuscitation in the acute phase and maintenance in the stable phase. Gu (7) and others showed that the rehabilitation exercise of early cardiac function can adjust the patient's body movement, which can repair the autonomic nerve function, thus promoting the patient's cardiac rehabilitation. However, in recent years, in the exercise rehabilitation treatment carried out in China, it has been found that there are still problems such as the lack of medical rehabilitation professional support, the lack of personalization of the existing exercise treatment program, and the fear of exercise expressed by patients (8–10); therefore, difficulties persist in the effective implementation of exercise rehabilitation treatment in patients with CHF (11, 12). Wang et al. (13) pointed out that although conventional exercise rehabilitation training can improve the cardiac function of patients to a certain extent, adverse events are prone to occur. Therefore, a safe and personalized nursing rehabilitation model that can comprehensively assess the early exercise rehabilitation of patients with CHF and meet their actual needs is needed in clinical nursing. To this end, the present study aims to explore the impact on patients with CHF using a refined nursing model, combined with early exercise rehabilitation, under a risk assessment strategy, which is reported as follows.

## 2 Information and methods

### 2.1 Clinical information

This study is experimental in nature, employing the formula for comparing the means of two independent samples in experimental research. The formula is $n1=n2=\frac{2{\left(Z_{\alpha }+Z_{\beta }\right)}^{2}\sigma^{2}}{\delta^{2}}$, where *n*₁ denotes the sample size of the control group, *n*₂ denotes the sample size of the study group, *σ* denotes the population standard deviation, *δ* denotes the difference between the two population means, and *Z_α_* and *Z_β_* denote the critical values of the standard normal distribution corresponding to *α* and *β*, respectively. This study selected *α* = 0.05 and *β* = 0.10. Preliminary trial results yielded *σ* = 6.10 and *δ* = 3.80. Consulted tables provided *Z_α_* = 1.645 and *Z_β_* = 1.282. Integrating all values into the formula determined a final sample size of 90 cases. This study enrolled 90 patients with CHF admitted to two wards of the Cardiovascular Department at our hospital between February 2024 and November 2024. The patients were randomly assigned using a random number table to either a control group or a study group, and were placed in two separate wards: one ward exclusively for the control group and the other exclusively for the study group. The study employed a blinded design; all enrolled patients remained unaware of their group assignment, and the researchers responsible for data collection, collation, and statistical analysis were also completely uninformed of the allocation scheme. The inclusion criteria were as follows: (1) meet the diagnostic criteria of chronic heart failure in China Heart Failure Diagnostic and Treatment Guidelines 2018 (4); (2) cardiac function grading II to III (New York Heart Association, NYHA grading); (3) no serious dysfunction of important organs (brain, liver, lungs, kidneys); (4) sound limb function; (5) clear consciousness and smooth communication; (6) low or medium risk of exercise; and (7) informed and voluntary participation in this study. The exclusion criteria were as follows: (1) patients with contraindications to exercise testing and training; (2) patients with end-stage disease or no voluntary activity; (3) those with cognitive dysfunction, communication disorders, and psychiatric disorders; (4) those who are currently participating in other interventional studies. Additional exclusion criteria: (1) patients or family members requesting to withdraw themselves; and (2) sudden illness, unable to continue to participate in the study. This study was approved by the Ethics Committee of Jiaxing Second Hospital (approval number 2024-089-01).

### 2.2 Methods

A comprehensive assessment of patients with CHF in the control group and the study group includes the following: (1) general data collection: through the questioning of patients with CHF and combined with the review of the patients' historical cases, fully understanding and recording the patients' complaints, disease history, other medical history, family history, lifestyle (smoking, alcohol consumption), compliance with the standardized use of heart failure medications and adverse drug reactions, and nutritional assessment, risk factors, etc. (2) Physical examination, vital signs monitoring, auscultation of heart sounds and lung rales, observation of jugular venous filling, hepatic jugular signs, and peripheral edema. (3) Assessment of cardiac function: B-type natriuretic peptide (BNP), left ventricular ejection fraction (LVEF), and 6-min walk distance (6-MWD). (4) Assessment of patients' ability to carry on their routine tasks by performing activities of daily living (ADLs), and assessment of patients' quality of life using the Minnesota living with heart failure questionnaire (MLHFQ).

The CHF patients in the control group underwent routine nursing care in the traditional mode. The following are the forms of oral teaching and distribution of teaching manuals and other teaching methods, including general care: dietary principles of low-salt diet, small and frequent meals, limiting sodium and water intake, remembering the amount of intake and output; rest and activities: reasonable arrangements for rest time, limiting physical activity, moderate exercise, avoiding exertion, avoiding colds; medication care: taking medication as prescribed, observing the role of medication and side effects; psychological care: ensuring adequate sleep, providing disease knowledge–related education, eliminating patients' concerns; quitting smoking and limiting alcohol, etc.

The patients with CHF in the study group applied the refined nursing model, combined with early exercise rehabilitation, under the risk assessment strategy on the basis of the control group: (1) formation of the CHF study group: (a) It consisted of the researcher herself, a cardiac rehabilitation doctor of the Department of Cardiology, a head nurse, a cardiac rehabilitation therapist, a cardiac rehabilitation nurse, two cardiovascular nurses, two data collection tools and recorders, and a data collation tool and statistician. (b) All members of the study group received learning and training on risk assessment, the refined nursing model, and theoretical knowledge related to early exercise rehabilitation in patients with CHF. Statisticians. (b) All members of the research team received study and training in risk assessment, the refined nursing model, and theoretical knowledge related to early exercise rehabilitation for patients with CHF. After training, team members selected patients for enrollment according to the nativity criteria, conducted assessments, and formulated plans and implemented interventions. The patients signed an informed consent form at the time of enrollment. (2) Implementation and application of the refined nursing model in early exercise rehabilitation of patients with CHF based on the risk assessment strategy: Phase 1: Standardized Assessment and Admission Screening (within 24 h) to identify patients suitable for early rehabilitation, ensure safety, and establish baseline data. The standardized assessment primarily encompasses the following: Collection of general patient details—physical examination—vital signs monitoring—cardiac function evaluation—assessment of ADLs and quality of life—evaluation of exercise habits and willingness to exercise—selection of the 6-min walk test (6MWT) as the exercise stress protocol (14)—Assessment of exercise risk in patients with CHF [low risk: 6MWD (15) >450 m, moderate risk: 6MWD 300–450 m; high risk: 6MWD <300 m; very high risk: 6MWD <150 m]. This study selected CHF patients classified as moderate or low risk for exercise. Phase 2: Refined early exercise rehabilitation nursing program development (refer to Chronic Heart Failure Cardiac Rehabilitation Expert Consensus 2020 Edition) (16): the general principles followed in the development of exercise prescription, including six elements: type of exercise, intensity, frequency, time, progress, and precautions, the specific content includes aerobic exercise, resistance exercise, flexibility training, and respiratory training (see Table 1 for details). Phase 3: Program Implementation and Safety Monitoring. Early exercise rehabilitation for CHF patients must adhere to the principle of “safety first, conservative progression.” Patients must comfortably complete the current intensity level (Borg RPE 11-13) in two consecutive training sessions without discomfort, while key physiological indicators remain stable: exercise heart rate increment <20 bpm, oxygen saturation >88%, blood pressure fluctuations within safe ranges, and rapid recovery of heart rate/shortness of breath within 5 min postexercise. Upon meeting these criteria, the rehabilitation program should progress sequentially: first extending duration, then increasing frequency, and finally elevating intensity. The exercise rehabilitation program must be conducted under “one-to-one supervision with continuous monitoring.” A cardiac rehabilitation therapist provides close guidance, continuously observing the patient's condition. An electrocardiogram monitor tracks the heart rate and blood oxygen levels in real time, with blood pressure measured every 5–10 min. Active questioning and communication ensure that the patient can engage in a brief conversation without any discomfort during exercise, enabling real-time assessment of their status. Clear indications for “immediate cessation of exercise” include the following: chest pain, severe arrhythmia, acute blood pressure abnormalities (systolic >220 mmHg or drop >10 mmHg with symptoms), SpO₂ plunge >5% or below 88%, and dizziness/syncope. Upon occurrence, exercise must be halted immediately, emergency services summoned, and the patient positioned for rest with oxygen administered. Subsequent documentation, analysis, and program reassessment are mandatory. Phase 4: Discharge Preparation and Long-Term Planning. Ensure patients transition smoothly to home or community-based rehabilitation, while maintaining long-term adherence. Conduct standardized assessments prior to discharge, such as 6MWD, LVEF, and MLHFQ, to quantify improvements in cardiac function and quality of life. Develop a discharge plan, provide written discharge instructions, and formulate a personalized home exercise program: recommend accessible, preferred activities (e.g., walking in local parks, practicing Tai Chi at home). Refer patients requiring ongoing rehabilitation to community cardiac rehabilitation centers; offer home-based rehabilitation services for those with mobility limitations. Conduct telephone follow-up within 24–72 h postdischarge.

**Table 1:** Refined early exercise rehabilitation nursing program.

Key constituent	Aerobics	Resistance movement	Flexibility training exercises	Breathing exercises
Kind	Walking, Baduanjin, Taijiquan, Square Dance, etc.	Small dumbbells, elastic bands, leg lifts, hip bridges, and other overcoming self-body mass exercises.	Power stretching, static stretching.	Lip-contraction breathing training, abdominal breathing training, artificial counter resistance breathing training.
Exercise intensity	Exercise intensity was calculated using the heart rate reserve method based on the 6MWT results: exercise target heart rate = %HRR (percentage of reserve heart rate) + resting heart rate, with the percentage set to gradually increase from 40%–80%.	Heart rate increase <20 beats/min, rating of perceived exertion (RPE) < 12.	3–5 general pulls on key muscle groups.	RPE < 12
Exercise time and frequency	The target levels were 20–60 min/session and ≥5 sessions/week, respectively, with 5–10 min of warm-up and finishing exercises included in the exercise period.	Upper-extremity muscle groups, core muscle groups (including chest, shoulders, upper back, lower back, abdomen, and glutes), and lower-extremity muscle groups are alternated on different days; 8–10 muscle groups are trained at a time, with the goal of 1–3 sets of each muscle group per workout, starting with 1 set of 10–15 repetitions per set in a progressive manner, with a rest interval of 2–3 min between sets. Each muscle group is trained 2–3 times per week, with a training interval of the same group of muscles at least 48 h.	Each lasts 20–30 s, after each resistance training session, or 2–3 times per week.	2–3 times a day, about 10 min each time 20–30 min per day, 5 times per week.
Movement progress	Adjusting exercise prescriptions to the 6MWT	Easily complete 3 sets and 10–15 repetitions per muscle group, increasing the number of repetitions per set (e.g., 15–25 repetitions per set) and decreasing the intensity of the training.	Gradually increase the number of pulls for muscle groups	Step by step
Exercise precautions	Evaluate and pay attention to the warmup and finishing phases, avoid the peak time of glucose-lowering medication blood concentration, increase the diet before, during, and after exercise to avoid hypoglycemia, pay great attention to the patient's complaints of discomfort and changes in signs during exercise, and make a good emergency plan	Adjust breathing patterns, avoid Valsalva maneuvers, do adequate preparation and organizing activities before and after exercise, monitor heart rate and blood pressure during exercise, start with low intensity, maintain correct posture, resistance training to not cause significant muscle pain is appropriate, if dizziness, palpitations or shortness of breath and other symptoms, stop exercising immediately!	According to the movement difficulty, amplitude, etc., step by step, according to the amount of strength, to avoid strains	Prevents dizziness, vertigo, and other discomforts associated with hyperventilation

### 2.3 Evaluation indicators

1. The evaluation of cardiac function in patients with CHF includes LVEF, BNP, and 6MWD. LVEF: data collected according to the report of cardiac ultrasound, with a normal range of 50%–70%; the lower the value, the lower the myocardial contractility and the worse the heart's contractile function. BNP: according to the report of laboratory examination, with a normal value of 0–100 ng/mL, the higher the value, the more serious the heart failure. 6MWD reference Standard (14): <150 m severe cardiac insufficiency, 150–425 m moderate cardiac insufficiency, 426–550 m mild cardiac insufficiency, the larger the value, the better the patient's cardiac function.
2. Assessment of daily life self-care ability (17) was assessed using the ADL scale with a full score of 100 points. A total score of ≤40 was classified as severe dependence, all of which required care by others; a total score of 41–60 was classified as severe dependence, most of which required care by others; a total score of 61–99 was classified as mild dependence, a small portion of which required care by others; and a total score of 100 was classified as no need for dependence and no need for care by others. The higher the score of ADL, the better the ability of the patient's daily living activities.
3. Quality of life assessment: assessed by MLHFQ (18) using a 6-point Likert scale from 0 to 5: 21 specification items, 0—none, 1—very slight, 2—slightly, 3—slightly obvious, 4—obviously, and 5—very obvious, with a range of scores from 0 to 105 points on three dimensions: symptomatic, physical activity, and affective, with higher scores implying a poorer quality of life for the patient.

### 2.4 Statistical methods

SPSS 24.0 statistical software was applied to analyze the data. Measurement data conforming to normal distribution were expressed as mean ± standard deviation (±s), an independent sample *t*-test was used for intergroup comparison, and a paired sample *t*-test was used for intragroup comparison; counting data were expressed as the frequency and constitutive ratio, and a *χ*^2^ test was used for comparison. A value of *P* < 0.05 was considered as the difference was statistically significant.

## 3 Results

A total of 90 cases of research subjects were initially included in this study, due to changes in condition during treatment, two patients in each of the control group and the research group withdrew from the study prematurely, and finally a total of 86 patients completed the study, 43 patients each in the control group and the research group. The general information of the two groups was compared, and the difference was not statistically significant (*P* > 0.05), and was comparable, see Table 2.

**Table 2:** Comparison of general information between the two groups.

Item		Control group (*n* = 43)	Research group (*n* = 43)	*t/x^2^*	*P*
Age (year)		72.63 ± 11.731	74.44 ± 10.072	0.769	0.444
Sex (%)	Men	21 (48.84)	24 (55.81)	0.420	0.666
	Women	22 (51.16)	19 (44.19)		
NYHA (%)	II	22 (51.16)	18 (41.86)	0.748	0.517
	III	21 (48.84)	25 (58.14)		
Education level (%)	Primary and lower	35 (81.40)	32 (74.42)	0.664	0.792
	Junior/senior/secondary	7 (16.28)	10 (23.26)		
	College and above	1 (2.33)	1 (2.33)		

The LVEF and BNP of the two groups, and the 6MWD of the study group were compared ($\bar{\chi }\pm s$) and the differences were statistically significant (*P* < 0.05), as given in Tables 3, 4.

**Table 3:** Comparison of LVEF and BNP between the two groups ($\bar{\chi }\pm s$).

Item	Control group (*n* = 43)	Research group (*n* = 43)	*t*	*P*
LVEF (%) precare	48.51 ± 13.537	42.86 ± 14.897	1.841	0.069
Aftercare	47.60 ± 11.881	53.21 ± 12.045	−2.172	0.033
*t*	0.330	−3.542		
*P*	0.742	<0.001		
BNP (ng/L) precare	810.2212 ± 657.16160	1,053.7012 ± 911.43855	−1.421	0.159
Aftercare	486.0358 ± 454.51159	312.8286 ± 294.78658	2.097	0.039
*t*	2.661	5.072		
*P*	0.009	<0.001		

**Table 4:** Comparison of 6MWD in study groups ($\bar{\chi }\pm s$).

Item	Control group (*n* = 43)	Research group (*n* = 43)	*t*	*P*
6MWD (m) precare	346.40 ± 24.376	344.86 ± 27.306	0.275	0.784
Aftercare	359.86 ± 23.861	394.05 ± 42.537	−4.594	<0.001
*t*	−2.589	−6.381		
*P*	0.011	<0.001		

A comparison of ADL between the two groups ($\bar{\chi }\pm s$) showed that the difference was statistically significant (*P* < 0.05), see Table 5.

**Table 5:** Comparison of ADLs between the two groups ($\bar{\chi }\pm s$).

Item	Control group (*n* = 43)	Research group (*n* = 43)	*t*	*P*
ADL (score) precare	74.77 ± 8.518	77.56 ± 12.648	−1.200	0.233
Aftercare	85.23 ± 7.864	92.91 ± 9.712	−4.027	<0.001
*t*	−5.919	−6.312		
*P*	<0.001	<0.001		

The MLHFQ of the two groups was compared ($\bar{\chi }\pm s$), and the difference was statistically significant (*P* < 0.05), see Table 6.

**Table 6:** Comparison of the MLHFQ between the two groups ($\bar{\chi }\pm s$).

Item	Control group (*n* = 43)	Research group (*n* = 43)	*t*	*P*
MLHFQ (分) precare	38.00 ± 18.731	41.35 ± 15.074	−0.913	0.364
Aftercare	30.81 ± 15.938	24.35 ± 10.863	2.198	0.031
*t*	1.916	6.000		
*P*	0.059	<0.001		

## 4 Discussion

### 4.1 Refined care model under the risk assessment strategy, combined with early exercise rehabilitation, improves cardiac function in patients with CHF

In this study, the percentage of LVEF in the study group increased significantly compared with that in the control group, the BNP value decreased significantly compared with that in the control group, and the 6MWD in the study group increased significantly after care compared with before care (*P* < 0.05), all of which was statistically significant, suggesting that the refined care model under the risk assessment strategy, in conjunction with early exercise rehabilitation, improves the cardiac function of patients with CHF, which may be related to the implementation of early exercise rehabilitation in these patients. The Heidenreich et al. (19) study, which deals with exercise-based cardiac rehabilitation for the improvement of cardiac function in patients with heart failure, is recommended as Class I evidence level A. Early exercise rehabilitation can effectively promote limb muscle contraction and skeletal muscle blood flow velocity, speed up the reversal of the symptoms of myocardial damage, and accelerate the rate of cardiac function recovery, and risk assessment is a prerequisite for and a guarantee of patients' cardiac rehabilitation, as it involves a comprehensive evaluation of the patient's condition to identify potential risks, followed by the implementation of appropriate measures to mitigate or eliminate these risks, thereby ensuring the safety of the rehabilitation process. The results of one study (20) showed that impaired HRR1 (1-min postexercise heart rate decline value) increased the risk of all-cause mortality by 70% (HR = 1.70), highlighting the central role of dynamic risk assessment in rehabilitation. It is a scientific approach that can help people to better understand and manage all types of risk. In this study, the research group conducted a comprehensive risk assessment of early exercise rehabilitation for patients with CHF and applied 6MWT to select CHF patients with a low degree of exercise risk whose risk stratification was medium or low risk, which avoided the adverse events caused by early exercise rehabilitation The exercise rehabilitation could be implemented safely without any events, which is in line with the findings of scholars such as Sun et al. (21), Liu et al. (22), and so on. The use of the risk-assessed exercise rehabilitation nursing model can improve the cardiac function of patients with CHF, which is consistent with the findings of Rongmei (23) and Song et al. (24). Refined nursing, on the other hand, is based on the traditional nursing model to specify, detail, and standardize the nursing content, constantly summarize the clinical nursing experience, break down the nursing procedures in fine detail, and implement targeted and personalized quality nursing services, which can better improve the prognosis of patients. A number of studies (25, 26) have confirmed the application of refined nursing care in surgical patients and its facilitating effect on the recovery of postoperative patients, as well as an improvement in patients' psychological status. In this study, the research group formulated a personalized early exercise rehabilitation nursing program for patients with CHF according to their aerobic exercise, resistance exercise, flexibility training, and respiratory training exercise prescription, which refined the exercise rehabilitation nursing link, improved patient adherence, and could lead to a better implementation of the exercise rehabilitation and an improvement in the patients’ cardiac function. Therefore, the refined nursing care model under the risk assessment strategy, combined with early exercise rehabilitation, can improve the cardiac function of patients with CHF.

### 4.2 Refined care model under the risk assessment strategy, combined with early exercise rehabilitation, improves the quality of life of patients with CHF

In this study, the ADL value of the study group increased significantly compared with that of the control group, and the MLHFQ value decreased significantly compared with that of the control group (*P* < 0.05), which are statistically significant, indicating that the refined nursing model under the risk assessment strategy, combined with early exercise rehabilitation, can improve the quality of life of patients with CHF. This may be related to the fact that the refined nursing model under the risk assessment strategy can improve the process of implementation of early exercise rehabilitation. This, in turn, may be related to the fact that early motor rehabilitation can improve the process of implementation of early motor rehabilitation. Through early motor rehabilitation, nerve excitation can be transmitted to the central nervous system, which can form a sensory stimulation of varying depths and shades, release a large number of nerve impulses, accelerate the reorganization of nerve cells, speed up the formation of collateral circulation, activate the neural pathways in the latent state, play its own role in plasticity, and improve the level of nerve growth factors in the intracranial cortex and hippocampus, which will ultimately promote the restoration of limb function (27). Relevant studies have shown (28) that exercise rehabilitation is conducive to increasing the level of secretion of doxylamine and other substances in the brain and improving the emotional and psychological states of patients, thereby accelerating the recovery of the condition and improving their overall physical and mental health. At the same time, cardiac rehabilitation can improve the clinical prognosis of patients with heart failure, reduce the readmission and mortality rates, and improve the quality of life of patients. The use of rehabilitation training can help change the patient's passive training regimen to an active one. Applying the refined nursing model under the risk assessment strategy will help to adjust the previous traditional training program, which is more targeted, increase the levels of the patient's acceptance and cooperation with exercise, reduce the patient's anxiety and fear, promote the recovery of the heart pump function, and decrease the number of adverse cardiovascular events (29), all of which will further enhance the patient's daily life activity, exercise endurance, and the psychological state, raising hopes for leading a better life in the future.(30, 31). Therefore, it can be concluded that the refined care model under the risk assessment strategy, combined with early exercise rehabilitation, can improve the quality of life of patients with CHF.

## 5 Conclusion

In summary, refined nursing care under the risk assessment strategy is a prospective nursing intervention method that assesses the possible risks of patients, determines the relevant nursing risk factors and the degree of risk, and then provides hierarchical nursing care for the patients to ensure that they can get the appropriate interventions, interventions that are more precise, scientific, and targeted. Such a refined nursing care will help effectively avoid the pitfalls of conventional nursing(32). The risk assessment strategy can predict the possible adverse events that may occur in the process of rehabilitation and exercise, and the refined nursing care can formulate personalized and practical nursing intervention plans for patients through a comprehensive and detailed assessment of patients. Therefore, guided by risk assessment strategies, the refined nursing model has standardized and personalized the implementation of early exercise rehabilitation measures. This has enhanced the acceptance, cooperation, and adherence to exercise among patients with CHF while simultaneously reducing the incidence of adverse events. This model effectively safeguards the safety and implementation outcomes of early exercise rehabilitation in patients with CHF. Modifying the physiological mechanisms (see Discussion sections 4.1 and 4.2) improves cardiac function and elevates patients' quality of life.

### 5.1 Limitations of this study

The sample size in this study was small, the intervention time was short, and none of the patients were involved in follow-up. In the future, the sample size can be expanded, and patients can be followed up regularly after discharge to prolong the intervention time and observe the long-term clinical effects to further confirm the findings of this study.

## Data Availability

The original contributions presented in the study are included in the article/Supplementary Material, further inquiries can be directed to the corresponding author.
